# Health, Heredity and Personal Beauty

**Published:** 1891-05

**Authors:** J. C. Elson


### Book Reviews.

HEALTH, HEREDITY AND PERSONAL BEAUTY By J. V. Shoemaker, A. M., M. D. F. A. Davis, publisher, Philadelphia.

Those especially interested in physical culture will be greatly entertained and much benefitted by a perusal of Dr. Shoemaker's "Health, Heredity and Personal Beauty." It contains many interesting facts and useful hints, and is withal so gracefully and pleasingly written as to chain the attention of the reader from the outset.

While the work is, strictly speaking, a scientific one, yet it can be read with pleasure and profit by any. It is as interesting as a novel. Not only does Dr. Shoemaker present many plain and important facts as to cleanliness, the cosmetic care and treatment of the face, but he also gives many pleasing theories of the more æsthetic side of physical culture.

His chapter on the "Art of Walking" is admirable, and urges the importance of the greater cultivation of an easy and graceful carriage. He claims that "grace is fundamentally beauty of movement in living or life-like things," and in very admirable language presents his theories and ideas on interesting topics. Altogether, the book is a valuable one, and can be read profitably alike by the doctor and his patient.

J. C. ELSON, M. D.

